# Can electronic cigarettes help pregnant smokers quit, and are they as safe to use in pregnancy as nicotine replacement treatments?

**DOI:** 10.1002/ctm2.1064

**Published:** 2022-09-20

**Authors:** Francesca Pesola, Anna Phillips‐Waller, Dunja Przulj, Katie Myers Smith, Peter Hajek

**Affiliations:** ^1^ Wolfson Institute of Population Health Barts and The London School of Medicine and Dentistry Queen Mary University of London London UK

**Keywords:** E‐cigarettes, pregnancy, safety, smoking cessation

Smoking during pregnancy is associated with a number of adverse pregnancy outcomes.^1^ Helping pregnant smokers quit is thus important. However, stop‐smoking treatments such as nicotine replacement therapy (NRT) and bupropion show only limited efficacy in this population^2^, ^3^, ^4^ while the third licensed stop‐smoking medication, varenicline, is not recommended in pregnancy.

Electronic cigarettes (ECs) are a form of NRT that seems to be more effective than other forms of NRT.^5^, ^6^, ^7^ ECs are, however, a consumer product rather than a licenced medication and there are concerns that they may carry a degree of risk, although this is expected to be very small compared to risks of smoking.^7^

## 1 WHAT ARE THE SAFETY ISSUES?

Nicotine may pose risks to the developing fetus, although the evidence of this is only available from animal studies with forced chronic high doses of nicotine.^8^ Such effects have not been documented for nicotine doses taken voluntarily by humans.^3^, ^9^ Indeed, NRT products to help pregnant smokers quit are approved by licensing authorities in most countries. EC aerosol, however, contains other chemicals in addition to nicotine and objective data on pregnancy outcomes in women who switch from smoking to EC use are urgently needed.

We carried out the first randomised controlled trial that compared the effectiveness and safety of nicotine patches (combined with other NRT products if required) versus ECs in 1,140 pregnant smokers.^10^

## 2 DID E‐CIGARETTES HELP PREGNANT SMOKERS QUIT?

Based on the primary outcome (validated prolonged abstinence at the end of pregnancy), the proportion of women who quit smoking was low and similar in the two study arms (EC = 6.8% vs. NRT = 4.4%). However, validation of self‐reported abstinence was done via postal saliva samples. Women in late pregnancy or with newborn babies may have found going through the instructions and shipping the samples difficult, as only 55% of women reporting abstinence provided useable samples. Self‐reported abstinence at end of pregnancy (as opposed to validated prolonged abstinence from the start of the trial) was significantly higher in the EC arm (20.7% versus 13.7%, *p* = 0.002).

We were also concerned that ECs would be more attractive to pregnant smokers than patches and that more women allocated to the patch arm would stop smoking with the help of ECs than women allocated to the EC arm quitting with NRT. We pre‐specified a sensitivity analysis that excluded these participants and this showed 6.8% versus 3.6% (EC vs. NRT; *p* = 0.02) validated prolonged abstinence and 19.8% versus 9.7% (EC vs. NRT; p < 0.001) self‐reported abstinence at the end of pregnancy.

The unadjusted primary analysis thus provided insufficient evidence to confidently demonstrate that ECs are more effective than NRT. The effect of ECs appears to have been masked by EC use in the NRT arm. When abstinent participants using non‐allocated products were excluded, ECs were markedly more effective than patches in all abstinence outcomes.

## 3 WERE E‐CIGARETTES AS SAFE AS PATCHES?

The mean birthweight and rates of adverse birth outcomes were similar in the two study arms, with one exception. There were more babies with low birth weight (<2.5 kg) in the group allocated to NRT use.

This could be a chance finding, but in a previous large study that compared nicotine and placebo patches, the nicotine arm had better birth and infant outcomes than the placebo arm 2 years after delivery.^3^ Both findings are likely the result of a larger reduction in smoking in the study arms with more favourable safety outcomes.

EC use in pregnancy does not seem to pose larger risks than the use of NRT, despite the fact that ECs were more likely to be used and were used for longer periods than NRT.

## 4 CONCLUSIONS

ECs might help women who smoke while pregnant to stop smoking. Their use during pregnancy appears to be as safe as the use of NRT and may reduce the risk of having a low‐birth‐weight baby compared to the use of NRT. In countries where stop‐smoking advice includes, among other treatment options, a recommendation to switch to ECs, such a recommendation can be extended also to pregnant smokers.

## CONFLICT OF INTEREST

PH provided consultancy to and received research funding from Pfizer. DP received research funding from Pfizer. The remaining authors have no conflicts of interest to declare.
